# YuNü-Jian attenuates diabetes-induced cardiomyopathy: integrating network pharmacology and experimental validation

**DOI:** 10.3389/fendo.2023.1195149

**Published:** 2023-05-23

**Authors:** Wei Wang, Ruixia Liu, Yingying Zhu, Lina Wang, Yu Tang, Baolei Dou, Shuo Tian, Furong Wang

**Affiliations:** ^1^ College of Traditional Chinese Medicine, Shandong University of Traditional Chinese Medicine, Jinan, China; ^2^ Department of Geriatric Medicine, Affiliated Hospital of Shandong University of Traditional Chinese Medicine, Jinan, China

**Keywords:** diabetic cardiomyopathy, molecular docking, network pharmacology, Nrf2, NQO1, SIRT1, YuNü-Jian

## Abstract

**Introduction:**

Diabetic cardiomyopathy (DCM) is one of the most prevalent complications of diabetes with complex pathogenesis. YuNü-Jian (YNJ) is a traditional Chinese medicinal formula widely used for diabetes with hypoglycemic and cardioprotective effects. This study aims to investigate the actions and mechanisms of YNJ against DCM which has never been reported.

**Methods:**

Network pharmacology approach was used to predict the potential pathways and targets of YNJ on DCM. Molecular docking between hub targets and active components of YNJ was performed and visualized by AutoDock Vina and PyMOL. Then type 2 diabetic model was employed and intervened with YNJ for 10 weeks to further validate these critical targets.

**Results:**

First, a total of 32 main ingredients of YNJ were identified and 700 potential targets were screened to construct herb-compound-target network. Then 94 differentially expressed genes of DCM were identified from GEO database. After that, PPI network of DCM and YNJ were generated from which hub genes (SIRT1, Nrf2, NQO1, MYC and APP) were assessed by topology analysis. Next, functional and pathway analysis indicated that the candidate targets were enriched in response to oxidative stress and Nrf2 signaling pathway. Furthermore, molecular docking revealed strong affinity between core targets and active components of YNJ. Finally, in rats with type 2 diabetes, YNJ obviously attenuated cardiac collagen accumulation and degree of fibrosis. Meanwhile, YNJ significantly upregulated protein expression of SIRT1, Nrf2 and NQO1 in diabetic myocardium.

**Discussion:**

Collectively, our findings suggested that YNJ could effectively ameliorate cardiomyopathy induced by diabetes possibly through SIRT1/Nrf2/NQO1 signaling.

## 1 Introduction

Diabetic cardiomyopathy (DCM) refers to diabetes-induced myocardial structural/functional abnormalities in the absence of coronary heart disease, hypertension, and valvular heart disease. The progressive pathophysiological changes of DCM consist of myocardial remodeling, diastolic/systolic dysfunction, heart failure, and even sudden death (1). According to the data from IDF, the global prevalence of diabetes is estimated to be 12.2% (783.2 million) in 2045 (2), among which approximately 11.7% to 67% develop DCM in the most and least restrictive criteria, respectively (3, 4). Therefore, seeking available strategies to reduce the susceptibility and intervene the development of DCM is of enormous clinical and social values.

At present, management of DCM mainly focuses on lowering blood glucose and lipid levels with no reliable specific drugs. However, whether the diabetic patients developed diastolic dysfunction and progressed to heart failure was independent of HbA1c levels, blood pressure, and lipid control (5). Newer glucose-lowering drugs such as GLP-1 agonists, DPP-4 inhibitors, and SGLT2 inhibitors have shown some cardioprotective effects (1). However, meta-analysis found that some drugs did not significantly improve cardiac structural abnormalities (6), and some did not reduce the risk of heart failure in patients with type 2 diabetes (7). This may be due to the complicated mechanisms involved in DCM. Thus, comprehensive medications such as Chinese Medicine may be another potential regimen to counteract DCM.

In traditional Chinese medicine, symptoms associated with DCM have been depicted as early as 1,400 years ago. According to the theory of Chinese medicine, the basic pathogenesis of DCM is dry heat and yin deficiency, which runs through the whole process of DCM (8). Thus, herbs and compound prescriptions with heat-clearing and yin-supporting efficacies can be effective interventions for DCM (8). YuNü-Jian (YNJ) is a traditional Chinese medicinal formula recorded in the medical classic Chinese book Jingyue`s Complete Works (Jingyue Quanshu). The main ingredients of YNJ are Gypsum (Shi Gao), Rehmanniae Radix Praeparata (Di Huang), Anemarrhenae Rhizoma (Zhi Mu), Ophiopogonis Radix (Mai Dong), and Achyranthis Bidentatae Radix (Niu Xi). This compound has been extensively used for the treatment of diabetes by clearing stomach heat and nourishing kidney yin (9). Recent experiments have found that YNJ protected pancreatic islet function and reduced blood glucose level under diabetes by regulating autophagic apoptosis of β-cells (10) and promoting gastric emptying (11). In addition, YNJ could improve ventricular remodeling after myocardial infarction induced by coronary artery ligation (12). These effects offer the possibility of YNJ for prevention and treatment of DCM. However, no relevant reports have been conducted so far.

Chinese medicine formulations are characterized by diverse components and synergistic effects acting through orchestrated biological processes, which makes the study of the mechanisms and actions of herbal formulas difficult. Network pharmacology offers favorable opportunity to assess the possible mechanisms underlying the observed clinical effects of herbal medicines. By constructing a component–target–gene network and performing a series of topological analyses, network pharmacology predicts therapeutic pathways and targets for subsequent validation by *in vivo* and *in vitro* experiments. This makes it possible to investigate the interaction between herbal formulations and disease with multifactorial pathogenesis, which cannot be readily verified by conventional experimental modes based on the “one gene, one drug, one disease” paradigm (13).

**Figure 1:**
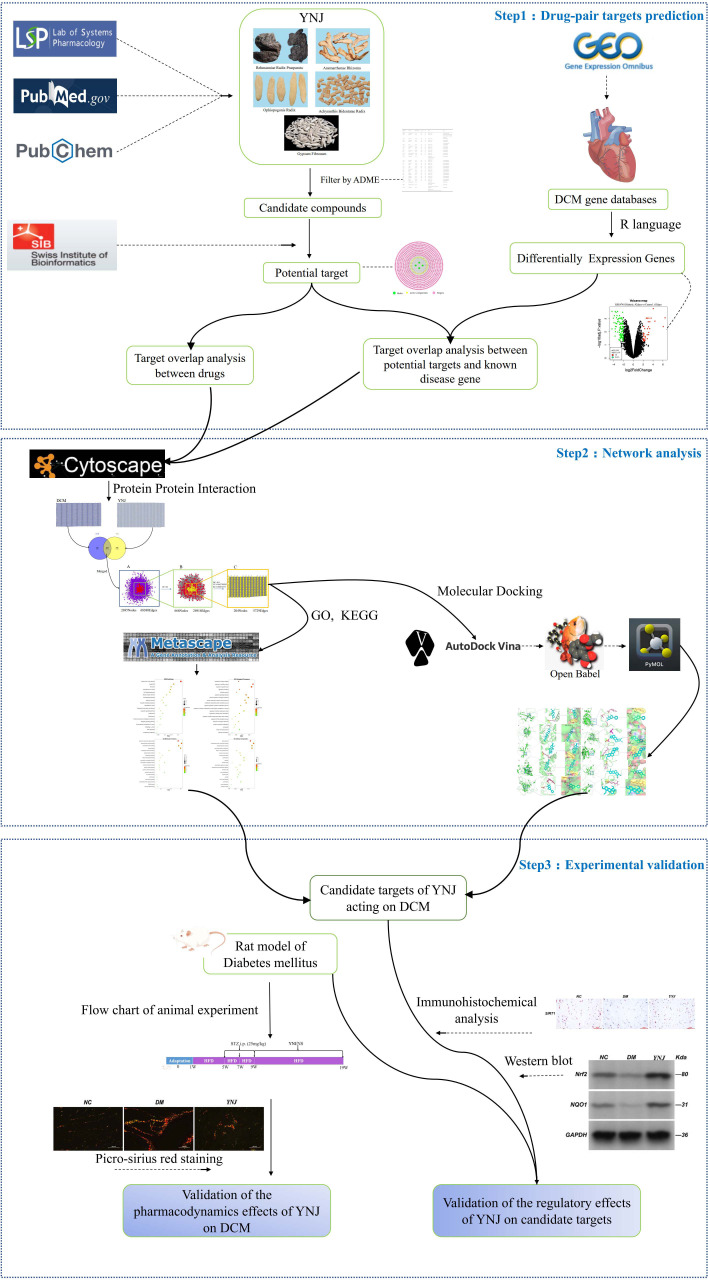
Flow diagram of bioinformatics combined with experimental pharmacology for investigating the effects and mechanisms of YNJ in the treatment of DCM.

In this study, network pharmacology techniques were applied to explore the potential mechanisms of YNJ on DCM, including identification of active components in YNJ, retrieval of DCM differentially expressed genes (DEGs) in the GEO database, drug target prediction, PPI network construction and topology analysis, KEGG pathway analysis, and GO biological analysis. Then, the molecular docking between the active ingredients and the predicted targets was carried out. Finally, a diabetic animal model was established to verify the improvement of cardiomyopathy by YNJ and the potential mechanism through the putative targets. The detailed strategy of this study is summarized in **Figure 1**.

## 2 Materials and methods

### 2.1 Collection of active compounds of YNJ and their potential targets

The chemical components of each traditional Chinese medicine in the YNJ decoction were searched on the TCMSP (https://tcmsp-e.com/) platform. The oral bioavailability (OB) ≥ 30% and drug-likeness (DL) ≥ 0.18 of the absorption, distribution, metabolism, and excretion (ADME) parameters were used as the criteria for screening. In order to ensure the integrity and reliability of the study, active ingredients were supplemented through literature review. Those with high contents and explicit pharmacological effects but not in accord with the above criteria were also considered as active candidate ingredients (14–17).

The screened Pubchem CID was input into the PubChem compound database (https://pubchem.ncbi.nlm.nih.gov/) database, and the SMILES number corresponding to each compound was obtained after eliminating compounds that did not match those in PubChem. Then, the Swiss Target Prediction database (http://www.swisstargetprediction.ch/) was used to predict the targets of active compounds (18). Finally, the UniProt ID of the target was searched through the Universal Protein Resource (UniProt) database (https://www.uniprot.org/) for batch standardization, with species set to *Homo sapiens*.

### 2.2 Construction of the active component–target network of YNJ

The network mapping software Cytoscape (Version 3.8.2) was applied to construct a network of active ingredients of YNJ and their targets. In the network diagram, a node represents an herb, an active ingredient, or a target, and the edge represents the interaction between them. The degree value of a node indicates the number of edges between the nodes in the network; the larger the value, the more likely the target is to be the key target of compounds.

### 2.3 Acquisition of differentially expressed genes of DCM

The gene expression datasets analyzed in this study were obtained from the GEO database (https://www.ncbi.nlm.nih.gov/geo/), from which the GSE4745 dataset (GPL85 platform, [RG_U34A] Affymetrix Rat Genome U34 Array) was rigorously selected for further study. Samples from the GSE4745 dataset were grouped by R language (Version 4.1.0) to obtain significantly DEGs with adjusted *p* < 0.05 and |logFC| ≥ 2.0 as screening conditions. The DEGs were imported into the HCNC-Tools-HCOP (19) (https://www.genenames.org/tools/hcop/) online tool for species conversion, preserving the homologous genes of rat and human, which are the DCM-related genes of human to be studied.

### 2.4 Construction and analysis of the protein–protein interaction network

Protein–protein interaction (PPI) networks of YNJ targets and DCM-related DEGs were constructed and visually analyzed with the plug-in BisoGenet in Cytoscape software (Version 3.8.2). Core proteins were screened from the central network, which came from the intersection of the above two networks. The CytoNCA plug-in was adopted for multi-center topological parameter analysis and screening according to the degree sorting procedure. The core targets were evaluated according to three parameters: degree centrality (DC), closeness centrality (CC), and betweenness centrality (BC). The screening criteria were set as DC > 2 times of the median to obtain the network of core target from which the key genes for the treatment of DCM by YNJ were further identified with DC, CC, and BC > 1 time of the median.

### 2.5 GO and KEGG pathway enrichment analysis

Metascape (https://metascape.org) was employed to perform GO and KEGG signal pathway analysis with the aforementioned predicted targets. GO biological function analysis mainly describe the function of target genes, including molecular function (MF), cellular components (CC), and biological processes (BP). KEGG enrichment analysis interprets the signaling pathway enriched by the common genes of YNJ active components and DCM. Terms with *p* < 0.01, minimum gene overlap (Min Overlap) of 3, and minimum enrichment factor (Min Enrichment) > 1.5 were collected and grouped according to the similarity of their members. The top 20 crucially significant pathways were selected for further study and are displayed in the advanced bubble chart colored by − log10 (*p*-values).

### 2.6 Verification of the binding of active components of YNJ to target proteins by molecular docking

The 3D protein structure of key targets was downloaded from the RCSB Protein Data Bank (RCSB PDB, http://www.pdb.org/). After adding hydrogen and calculating charges, the crystal structure of core components was downloaded from PubChem. AutoDock Vina software was used to dehydrate and hydrotreat the receptor protein and conduct molecular docking. Afterwards, free energy of binding (in kcal/mol) was obtained as the indicator of the binding likelihood. The more the negative scoring (the greater the absolute value), the higher the binding force is between the compound and the protein. Generally, the binding energy ≤−5.0  kcal/mol can be considered as an excellent binding effect. Finally, PyMOL software was used for visualization.

### 2.7 Pharmacodynamic study of YNJ and experimental validation in diabetic rats

#### 2.7.1 Animals and induction of diabetes

Male SD rats aged 8 weeks with a body weight of 180–220 g were obtained from Beijing Charles River Laboratory Animal Technology Co., Ltd. (Beijing, China). All rats were housed in standard conditions with a 12-h light/dark cycle at 22 ± 2°C and a humidity of 55 ± 5% with free access to food and water. After adaptation for 1 week, animals were divided randomly into a negative control (NC) and a model group. Rats in the model group were fed with a high-fat diet (HFD) for 4 weeks and then intraperitoneally injected with 25 mg/kg freshly prepared streptozotocin (STZ, Sigma, USA) dissolved in citrate buffer (0.1 M, pH 4.5) three times every other week (20). Those in the NC group received continual standard diet and three intraperitoneal injections of citrate buffer alone. Three days after the last injection, rats with a fasting blood glucose of 11.1 mmol/L or higher were considered type 2 diabetes mellitus and subjected to subsequent experiments with persistent HFD feeding. All experimental protocols for animals conformed to the guidelines of National Institutes of Health, and were permitted by the Animal Care and Use Committee of Shandong University of TCM.

#### 2.7.2 Experimental protocol

The successfully induced rats were randomly divided into diabetic mellitus (DM) and YNJ-treated group (YNJ). The preparation of YNJ involved mixing of decocting-free granules of Gypsum Fibrosum, Rehmanniae Radix Praeparata, Anemarrhenae Rhizoma, Ophiopogonis Radix, and Achyranthis Bidentatae Radix (Tianjiang Pharmaceutical Co., Ltd, Jiangyin, Jiangsu, China) with distilled water to a final drug concentration of 0.9 g/ml. YNJ was administrated intragastrically at a dose of 4.52 g/kg every day according to the clinical effective dose for diabetic patients. Rats in the NC and DM groups received equal amount of 0.9% saline with the same administration route and duration. After treatment with YNJ or saline for 10 consecutive weeks, all rats were anesthetized and euthanized. Ventricular tissues were rapidly excised and fixed in 4% paraformaldehyde or frozen at −80°C for later assays.

#### 2.7.3 Picro-Sirius red staining

After fixation for 24 h, the cardiac tissues were dehydrated in ascending grades of alcohol and embedded in paraffin. Picro-Sirius red (PSR) staining was used to demonstrate cardiac fibrosis. Briefly, sections of 5 μm thickness were conventionally deparaffined with xylene and alcohol. Then, slides were incubated with PSR (S8060, Solarbio, Beijing, China) for 10 min and viewed using a Nikon Eclipse Ci-L microscope (Tokyo, Japan). The collagen volume fraction (CVF, %) within the tissue was identified and assessed with Image Pro Plus 6.0 software as the ratio of stained fibrillar collagen area to the view area. The CVF from six random fields (×200) were averaged and used to show the collagen deposition of this sample.

#### 2.7.4 Immunohistochemical staining

Paraffin-embedded sections of 5 μm thickness were kept at 60°C for 3 h in the oven and dewaxed with xylene and hydrated in gradient ethyl alcohol. The slides were subjected to microwave antigen retrieval treatment followed by endogenous peroxidase deactivation using 3% H_2_O_2_ for 10 min. After blocking of nonspecific binding with normal serum for 20 min, the sections were incubated with primary antibody against SIRT1 (8469, Cell Signaling Technology, Boston, USA) at 4°C overnight. The slides were then incubated with secondary antibodies at 37°C for 30 min, stained with DAB, counterstained with Mayer’s hematoxylin, and visualized by a highly sensitive inverted microscope (LV200, Olympus). Image analysis was performed by Image Pro Plus 6.0 software.

#### 2.7.5 Western blot analysis

Ventricular tissues were homogenized in lysis buffer and quantified for protein concentration with a commercial assay kit (Beyotime Biotechnology, Jiangsu, China). Equal quantities of proteins were separated on 10%–12% SDS-PAGE under denaturing conditions and transferred onto a PVDF membrane. The membrane was blocked using 5% nonfat milk in TBS containing 0.05% Tween 20 (TBST) for 2 h, and then immunoblotted overnight at 4°C with primary antibody, followed by incubation with HRP-conjugated secondary antibody for 1 h at room temperature. The immunoreactive proteins were visualized with an ECL-detection kit (Thermo Fisher Scientific, Pittsburgh, PA, USA) using a Tanon 6600 (Tanon, Shanghai, China) luminescent imaging workstation. Levels of protein were quantified using Image Pro Plus 6.0 software (Media Cybernetics, Rockville, MD, USA) for optical density values. All the target proteins were normalized by GAPDH and depicted as percentage of the NC. Rabbit anti-Nrf2 antibody (ab92946), rabbit anti-NQO1 antibody (ab34173), rabbit anti-GAPDH antibody (ab8245), and goat anti-rabbit IgG H&L (HRP) (ab6721) were all purchased from Abcam (Cambridge, UK).

#### 2.7.6 Statistical analysis

All data were expressed as mean ± SD and analyzed and plotted using GraphPad Prism 5 (Version 5.01). One-way analysis of variance (ANOVA) and Tukey’s test were used for statistical analyses. *p*-values less than 0.05 were considered to indicate statistically significant differences.

## 3 Results

### 3.1 Bioactive ingredients in YNJ

The candidate bioactive components of YNJ were screened out from the TCMSP database with OB ≥ 30% and DL ≥ 0.18. Together with literature retrieval, we obtained 2, 13, 4, and 17 bioactive ingredients from Rehmannia Radix Praeparata, Anemarrhenae Rhizoma, Ophiopogonis Radix, and Achyranthis Bidentatae Radix, respectively. After removing the duplicate values and those without targets, 32 bioactive ingredients were screened out and eventually included in the follow-up study (**Table 1**).

**Table 1:** Potential effective compounds of YNJ.

PubChem CID	Compound	Molecular Formula	OB(%)	DL	Filter by	Origin
12303645	3-Epi-beta-Sitosterol	C_29_H_50_O	36.91	0.75	OB ≥ 30% DL ≥ 0.18	Rehmanniae Radix Praeparata
5280794	Stigmasterol	C_29_H_48_O	43.83	0.76	OB ≥ 30% DL ≥ 0.18	Rehmanniae Radix Praeparata/Anemarrhenae Rhizoma/Ophiopogonis Radix/Achyranthis Bidentatae Radix
13855373	ZINC13374323	C_27_H_28_N_2_O_4_	58.02	0.52	OB ≥ 30% DL ≥ 0.18	Anemarrhenae Rhizoma
45270099	Mangiferolic acid	C_30_H_48_O_3_	36.16	0.84	OB ≥ 30% DL ≥ 0.18	Anemarrhenae Rhizoma
5280863	Kaempferol	C_15_H_10_O_6_	41.88	0.24	OB ≥ 30% DL ≥ 0.18	Anemarrhenae Rhizoma/Achyranthis Bidentatae Radix
5318980	Icaritin	C_21_H_20_O_6_	45.41	0.44	OB ≥ 30% DL ≥ 0.18	Anemarrhenae Rhizoma
21160900	Chrysanthemaxanthin	C_40_H_56_O_3_	38.72	0.58	OB ≥ 30% DL ≥ 0.18	Anemarrhenae Rhizoma
441594	Hippeastrine	C_17_H_17_NO_5_	51.65	0.62	OB ≥ 30% DL ≥ 0.18	Anemarrhenae Rhizoma
5318997	Icariin	C_33_H_40_O_15_	41.58	0.61	OB ≥ 30% DL ≥ 0.18	Anemarrhenae Rhizoma
6440659	n-cis-Feruloyltyramine	C_18_H_19_NO_4_	118.35	0.26	OB ≥ 30% DL ≥ 0.18	Anemarrhenae Rhizoma
99474	Diosgenin	C_27_H_42_O_3_	80.88	0.81	OB ≥ 30% DL ≥ 0.18	Anemarrhenae Rhizoma
13939145	cis-N-p-Coumaroyltyramine	C_17_H_17_NO_3_	112.9	0.2	OB ≥ 30% DL ≥ 0.18	Anemarrhenae Rhizoma
5372945	p-Coumaroyltyramine	C_17_H_17_NO_3_	85.63	0.2	OB ≥ 30% DL ≥ 0.18	Anemarrhenae Rhizoma
44575945	Timosaponin B II	C_45_H_76_O_19_			Anti-oxidant effects Inhibits cardiomyocyte apoptosis Cardioprotective effect (1)	Anemarrhenae Rhizoma
5283663	Chondrillasterol	C_29_H_48_O	42.98	0.76	OB ≥ 30% DL ≥ 0.18	Achyranthis Bidentatae Radix
5281325	Spinoside A	C_39_H_56_O_12_	41.75	0.4	OB ≥ 30% DL ≥ 0.18	Achyranthis Bidentatae Radix
27545171	ZINC17147377	C_27_H_44_O_7_	44.23	0.82	OB ≥ 30% DL ≥ 0.18	Achyranthis Bidentatae Radix
2353	Berberine	C_20_H_18_NO_4_^+^	36.86	0.78	OB ≥ 30% DL ≥ 0.18	Achyranthis Bidentatae Radix
72322	Coptisine	C_19_H_14_NO_4_^+^	30.67	0.86	OB ≥ 30% DL ≥ 0.18	Achyranthis Bidentatae Radix
5281703	Wogonin	C_16_H_12_O_5_	30.68	0.23	OB ≥ 30% DL ≥ 0.18	Achyranthis Bidentatae Radix
521367	delta(7)-Stigmastenol	C_29_H_50_O	37.42	0.75	OB ≥ 30% DL ≥ 0.18	Achyranthis Bidentatae Radix
5281605	Baicalein	C_15_H_10_O_5_	33.52	0.21	OB ≥ 30% DL ≥ 0.18	Achyranthis Bidentatae Radix
64982	Baicalin	C_21_H_18_O_11_	40.12	0.75	OB ≥ 30% DL ≥ 0.18	Achyranthis Bidentatae Radix
160876	Epiberberine	C_20_H_18_NO_4_^+^	43.09	0.78	OB ≥ 30% DL ≥ 0.18	Achyranthis Bidentatae Radix
222284	beta-Sitosterol	C_29_H_50_O	36.91	0.75	OB ≥ 30% DL ≥ 0.18	Achyranthis Bidentatae Radix
455251	Inophyllum E	C_25_H_22_O_5_	38.81	0.85	OB ≥ 30% DL ≥ 0.18	Achyranthis Bidentatae Radix
5281331	alpha-Spinasterol	C_29_H_48_O	42.98	0.76	OB ≥ 30% DL ≥ 0.18	Achyranthis Bidentatae Radix
19009	Palmatine	C_21_H_22_NO_4_^+^	64.6	0.65	OB ≥ 30% DL ≥ 0.18	Achyranthis Bidentatae Radix
5280343	Quercetin	C_15_H_10_O_7_	46.43	0.28	OB ≥ 30% DL ≥ 0.18	Achyranthis Bidentatae Radix
5319741	Methylophiopogonanone A	C_19_H_18_O_6_			Anti-oxidant effects Inhibits cardiomyocyte apoptosis (2)	Ophiopogonis Radix
46886723	Methylophiopogonanone B	C_19_H_20_O_5_			Anti-oxidant effects Protects HUVECs against H_2_O_2_-induced cell death (3)	Ophiopogonis Radix
46173859	Ophiopogonin D	C_44_H_70_O_16_			ROS scavenging Reduced oxidative stress Maintains Ca^2+^ homeostasis and reduces ER stress in cardiomyocytes (4)	Ophiopogonis Radix

### 3.2 Potential targets and the compound–target network of YNJ

According to the filters described above, 3,579 candidate genes that were targeted by YNJ were harvested from the Swiss Target Prediction database. Then 700 common targets were obtained by overlap analysis, showing synergistical effects among these bioactive components.

**Figure 2:**
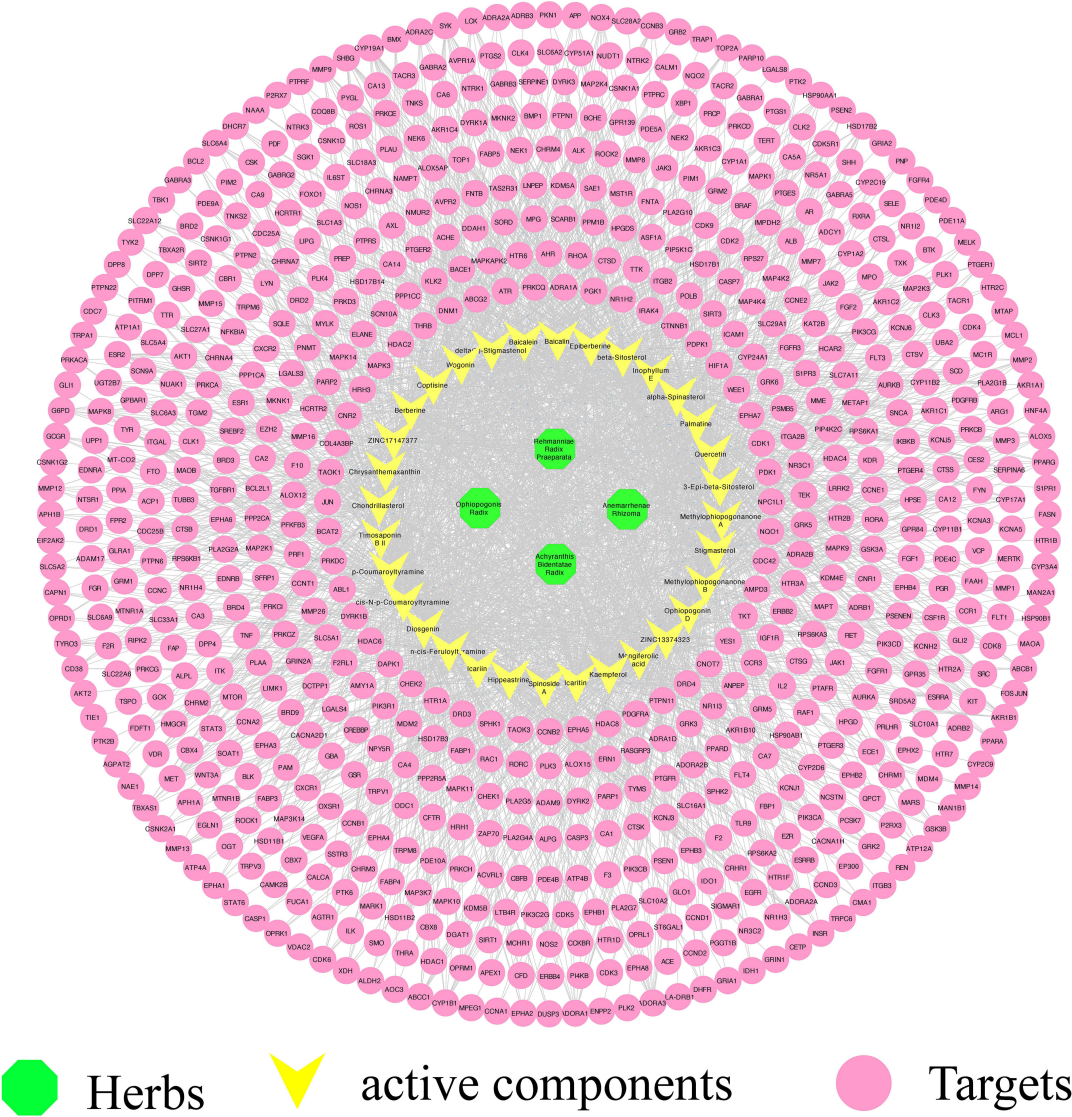
Effective component–target network of YNJ. The green nodes represent herbs, the yellow nodes represent active components, and purple nodes represent predicted targets.

By using Cytoscape, we constructed a compound–target network to identify the relationship between YNJ bioactive components and their candidate targets. The network was composed of 736 nodes and 2,504 edges (**Figure 2**). As revealed in the network, the average degree value of 32 active ingredients was 78.25, certifying the multi-target characteristics of YNJ. The average degree value of all compound targets was 3.53, suggesting systemic actions of diverse active components in YNJ. Moreover, all the four ingredients with highest degree values are from Anemarrhenae Rhizoma. This may be due to the twofold efficacies of clearing heat and nourishing yin, indicating the values of in-depth study aiming at this herb.

### 3.3 Identification of DEGs of DCM

**Figure 3:**
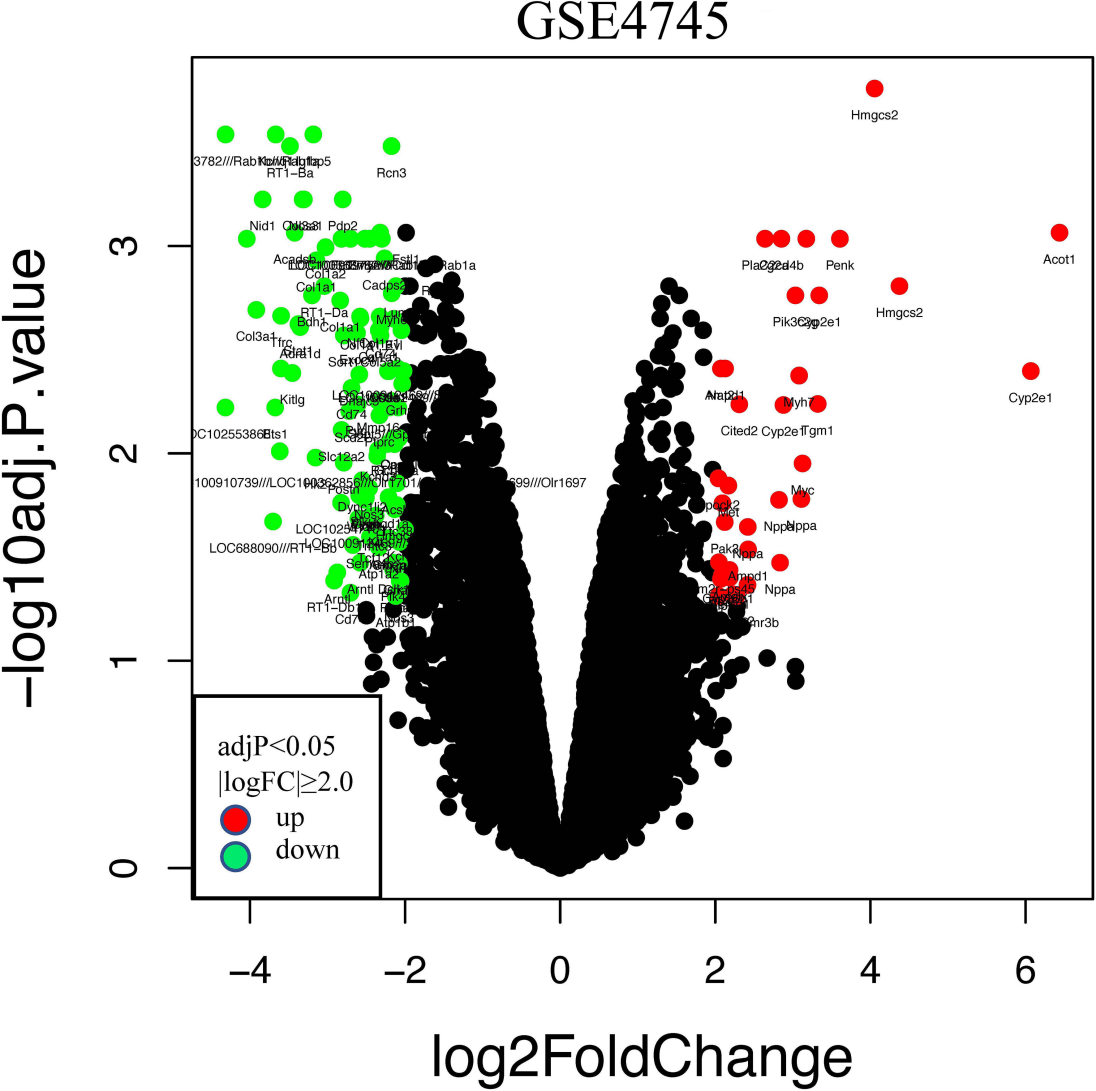
Volcano plots of differentially expressed genes (DEGs) in the GSE4745 dataset. Red and green plots represent up- and downregulated genes, respectively (|logFC| ≥ 2.0 and adjusted *p* < 0.05), while black represents no significant difference. FC, fold change.

The GSE4745 dataset was standardized and analyzed with the R package. According to the screening criteria of |logFC| ≥ 2.0 and adjusted *p* < 0.05, we obtained 113 DEGs, of which 26 were upregulated and 87 were downregulated. The related volcano map was created to show the distribution of these genes (**Figure 3**). Then, we converted these rat-derived genes into those of human by the HCNC-Tools-HCOP online tool. After getting rid of the ineligible genes, a total of 94 DEGs were obtained, with 24 upregulated and 70 downregulated.

### 3.4 PPI network analysis and hub gene identification

**Figure 4:**
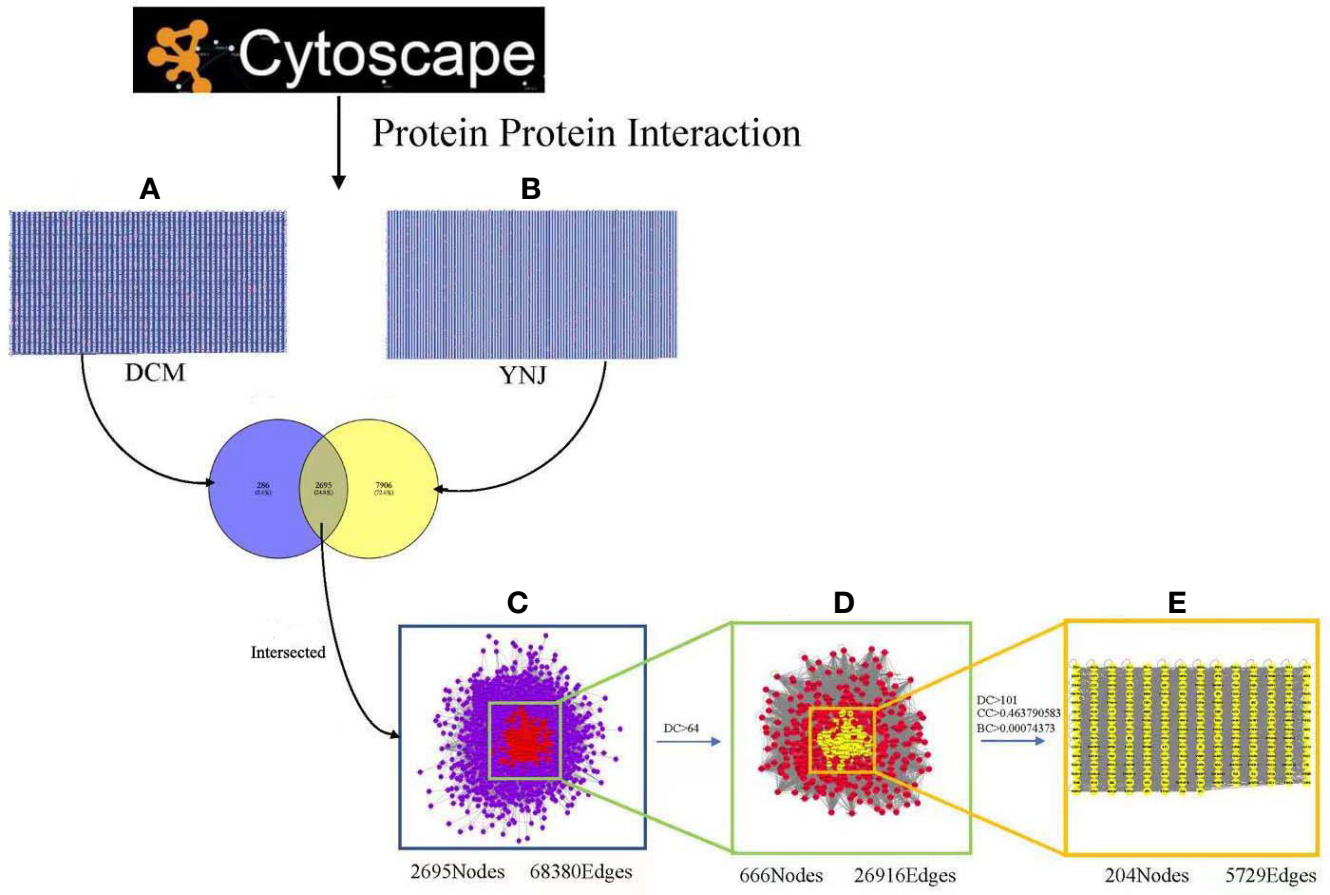
Identification of the core targets for YNJ against DCM. **(A)** The PPI network of DCM-related targets (2,981 nodes and 70,524 edges). **(B)** The PPI network of YNJ-related targets (10,601 nodes and 219,193 edges). **(C)** Intersection of PPI networks of YNJ putative targets and DCM-related targets (2,695 nodes and 68,380 edges). **(D)** PPI network of important targets obtained with the screening criteria of DC > 64 (666 nodes and 26,919 edges) from **(C)**. **(E)** Core–target PPI network of YNJ against DCM obtained with the screening criteria of DC >101, BC > 0.00074373, and CC > 0.463790583 (204 nodes and 5,729 edges) from **(D)**. BC, betweenness centrality; CC, closeness centrality; DC, degree centrality.

To further illustrate interactions between identified genes, the Cytoscape plugin BisoGenet was used to generate the PPI network of DCM and YNJ, respectively. The PPI network of DCM-related targets was constructed with 2,981 nodes and 70,524 edges, while the network of YNJ-related targets contained 10,601 nodes and 219,193 edges. Then, the two networks were merged into an intersection with 2,695 nodes and 68,380 edges. Afterwards, CytoNCA was used to assess the intersection of the PPI network by topological analysis. A network of YNJ against DCM, with 666 nodes and 26,919 edges, was first screened based on the criteria of DC > 64. A core–target PPI network was further screened with the criteria of DC >101, BC > 0.00074373, and CC > 0.463790583, and consisted of 204 nodes and 5,729 edges (**Figure 4**). The nodes with top degree values in the core–target PPI network are considered as hub genes, namely, SIRT1 (degree = 939), NQO1 (degree = 736), Nrf2 (degree =569), MYC (degree = 559), and APP (degree = 521).

### 3.5 GO functional enrichment and KEGG pathway analysis

**Figure 5:**
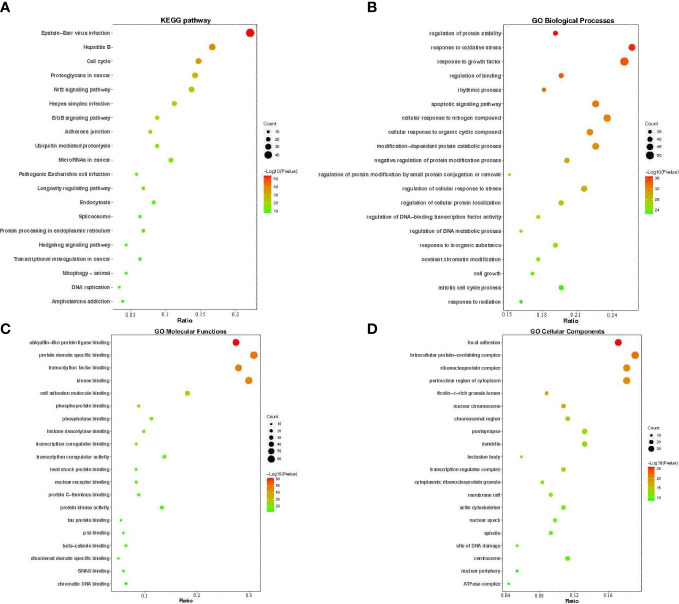
GO functional annotation and KEGG pathway enrichments. The bubble plots of the 20 most significant signaling pathways based on KEGG enrichment analysis **(A)**, and the top 20 enriched GO terms of biological process **(B)**, molecular function **(C)**, and cellular components **(D)**. The *X*-axis and *Y*-axis stand for the gene ratios and full names of the processes, respectively. The color and size of each bubble represent the *p*-value and gene count, respectively.

To further elucidate the mechanism of YNJ on DCM, GO function and KEGG pathway enrichment analysis aiming at 204 predicated key therapeutic candidates were performed in the Metascape platform. The top 20 significant GO terms during BP, CC, and MF, and the top 20 significantly enriched KEGG pathways were presented in the form of a bar and bubble diagram (**Figure 5**). BP that are involved in the treatment of YNJ on DCM were primarily associated with response to regulation to protein stability, response to oxidative stress, response to growth factor, regulation of binding, and so on. In the MF ontology, the targets of YNJ for DCM were mainly related to ubiquitin-like protein ligase binding, protein domain-specific binding, kinase binding, and so on. For the CC ontology, the targets were mainly involved in focal adhesion, intracellular protein-containing complex, ribonucleoprotein complex, perinuclear region of cytoplasm, and so on. KEGG enrichment analysis showed that the pathways significantly influenced by YNJ were cell cycle, proteoglycans in cancer, Nrf2 signaling pathway, ErbB signaling pathway, and so on. Therefore, it could be assumed that YNJ may exert protective effects on myocardium during diabetes mainly through modulation of oxidative stress, especially the Nrf2 pathway, which would be verified in the following docking analysis and *in vivo* experiment.

### 3.6 Molecular docking between hub targets and active components

**Figure 6:**
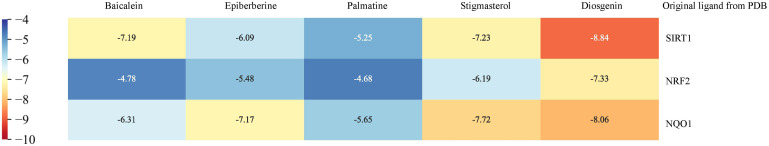
Binding affinity of key chemical compounds of YNJ to putative core targets as revealed by molecular docking.

**Figure 7:**
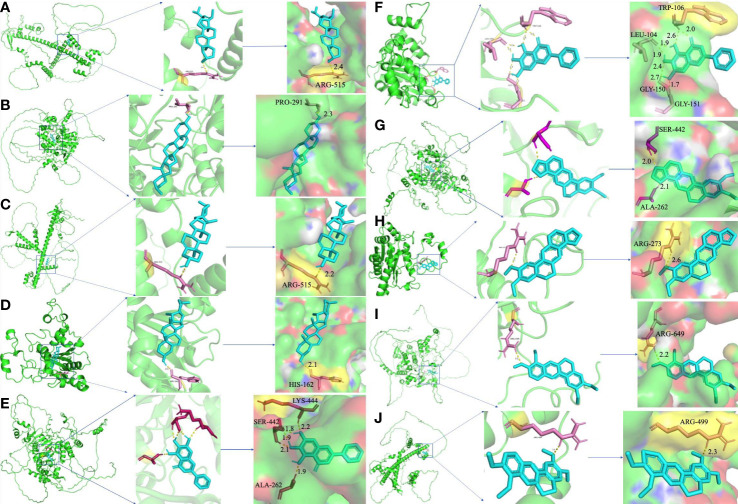
Molecular docking of hub targets and active components of YNJ. **(A)** Nrf2-Stigmasterol; **(B)** SIRT1-Diosgenin; **(C)** Nrf2-Diosgenin; **(D)** NQO1-Diosgenin; **(E)** SIRT1-Baicalein; **(F)** NQO1-Baiclein; **(G)** SIRT1-Epiberberine; **(H)** NQO1-Epiberberine; **(I)** SIRT1-Palmatine; **(J)** Nrf2-Palmatine.

To further certify the binding activity between the primary active ingredients of YNJ and key therapeutic targets, molecular docking with AutoDockTools was performed. The energy level represents their binding potency, with lower energy level indicating stronger binding capability. Generally, the binding affinity is considered to be potent if the docking calculation score (kcal/mol) is less than −7 in AutoDockTools. As shown in **Figure 6**, the calculated binding energies between main active components and key therapeutic targets were close to or even lower than −7, indicating specific binding possibility. Among them, diosgenin had the strongest binding affinity with DCM targets, as affinity with SIRT1 = −8.84 kcal/mol, with Nrf2 = −7.33 kcal/mol, with NQO1 = −8.06 kcal/mol. This supported the mechanistic involvement of the corresponding pathways in the effects of YNJ on DCM. In addition, as illustrated in **Figures 7B–D**, amino acid residue PRO-291 in the crystal structure of SIRT1, ARG-515 in Nrf2, and HIS-162 in NQO1 formed hydrogen bonds with diosgenin, respectively. **Figures 7A, E–J** shows the visualized images and details of optimal docking of key targets to other active components of YNJ.

### 3.7 Protective effects of YNJ on cardiomyopathy of diabetic rats

**Figure 8:**
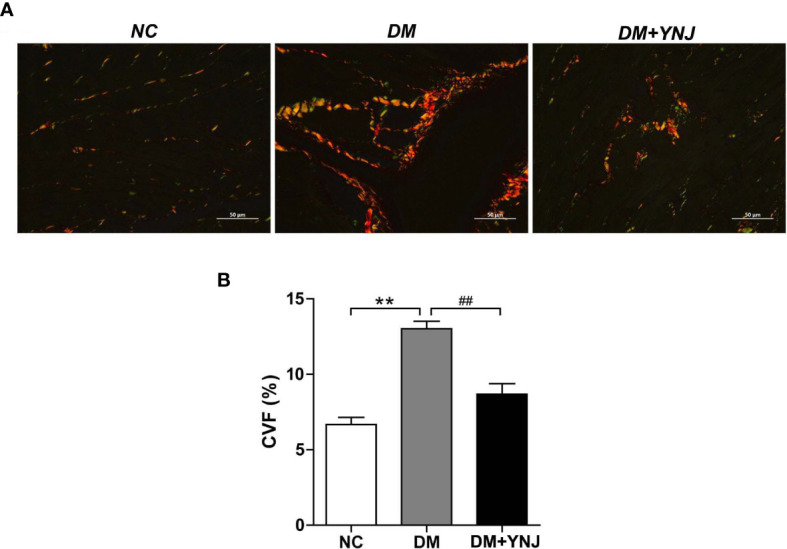
Picro-Sirius red (PSR) staining for collagen deposition in the myocardium. **(A)** PSR staining to detected collagen content and fibrosis in the left ventricle (400×). **(B)** Calculation of PSR-positive area as displayed by CVF. Data are presented as mean ± SD. **p  < 0.01 vs. the normal control group; ^##^ *p* < 0.01 vs. the DM group.

To further verify the cardioprotective effects of YNJ, diabetic rats were treated with YNJ for 10 weeks. Cardiac collagen accumulation and degree of fibrosis were evaluated by PSR staining. **Figure 8A** illustrates representative morphological changes showing a larger proportion of Sirius red collagen content in the myocardium of diabetic rats compared with controls. The collagen deposition as indicated by CVF significantly increased in the myocardium of rats with diabetes, which was markedly attenuated by YNJ administration (**Figure 8B**). The results implied that YNJ could ameliorate cardiac fibrosis induced by DCM, demonstrating cardioprotective effects.

### 3.8 Verification of critical targets of YNJ for DCM with animal experiments

**Figure 9:**
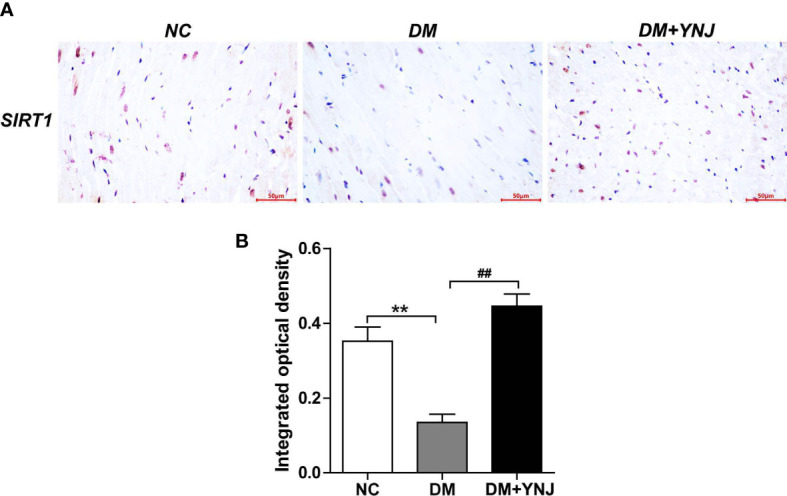
Immunohistochemical staining of SIRT1 in cardiac tissues. **(A)** Representative histological images of SIRT1 staining (400×). **(B)** Quantitative analysis of SIRT1 level. Data are expressed as mean ± SD. **p  < 0.01 vs. the normal control group; ^##^ *p* < 0.01 vs. the DM group.

**Figure 10:**
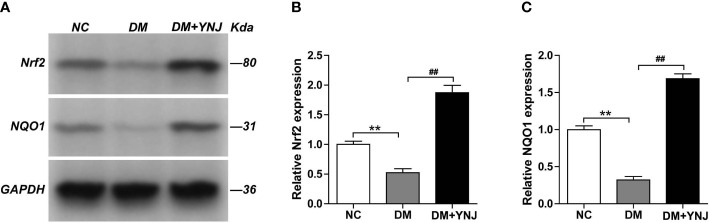
Western blotting analysis of Nrf2 and NQO-1 protein expressions in the myocardial tissues. **(A)** Representative blot images of Nrf2 and NQO-1 protein expressions in the myocardium. GAPDH served as the internal control. **(B, C)** Quantitative analysis of Nrf2 and NQO-1 protein levels. Data are presented as mean ± SD. **p  < 0.01 vs. the normal control group; ^##^ *p* < 0.01 vs. the DM group.

To clarify the effects of YNJ on the screened key targets against DCM, the expression of SIRT1, Nrf2, and NQO1 was detected by immunochemistry and Western blotting analysis. As shown in **Figures 9A, B**, diabetes induced the declined expression of SIRT1 in the cardiac tissues, which was reversed by YNJ administration. For Nrf2 and NQO1, we found significantly lower expression in the diabetic myocardium than the normal rats. YNJ treatment could effectively upregulate these oxidative-associated markers (**Figures 10A–C**). These results revealed the possible mechanisms of YNJ on cardiomyopathy initiated by diabetes.

## 4 Discussion

Although the treatment of DCM has been developed recently, practically effective interventions are scarce due to the complex etiology and pathogenic mechanisms of DCM. Based on the proprietary characteristics of multicomponents, multitargets, and multipathways, Chinese medicine has shown reliable therapeutic effects for DCM (21). In the present study, for the first time, we discovered the cardioprotective effects of YNJ in diabetic rats as reflected by alleviation of collagen deposition and fibrosis. Additionally, the PPI network of YNJ in the treatment of DCM was constructed, and pathway enrichment analyses were performed. More importantly, the key targets were screened and verified by molecular docking and *in vivo* experiments to illustrate the potential mechanisms of YNJ for DCM.

Cardiac remodeling is the major structural abnormalities of DCM. Here, in the myocardium of diabetic rats, we found obvious collagen deposition and fibrosis, which could be markedly reversed by YNJ administration. Similar protective function of YNJ on pathological remodeling has also been found in coronary artery ligation-induced myocardium infarction rats (12). Meanwhile, previous studies have found that effective components contained in YNJ possess apparent cardioprotective effects. For example, *Rehmanniae glutinosa*, the minister medicine in YNJ, attenuated adriamycin-induced cytotoxicity and apoptosis in H9C2 cardiac muscle cells (22). Timosaponin B, one of the primary bioactive compounds from *Anemarrhena asphodeloides*, attenuated isoproterenol-induced myocardial injury by inhibiting ER stress-mediated apoptosis pathways (14). In combination with these observations, our results highlight the cytoprotective property of YNJ in diabetic rats. Therefore, the underlined mechanism was next detected.

It is well-documented that oxidative stress characterized by excessive ROS production is one of the leading factors in the development of DCM. The key pathological processes underlying cardiac remodeling in diabetes are highly redox sensitive, including cardiomyocyte hypertrophy, apoptosis, fibrosis, and diastolic/systolic dysfunction (23). Oxidative stress and other risk factors may promote cardiomyocyte death, interstitial fibrosis, and cardiac stiffness, leading to diastolic and systolic dysfunction, and eventually heart failure. Therefore, pharmacological strategies for reducing ROS burden or increasing antioxidant mechanisms may provide a successful strategy for the treatment of DCM (24). Here, the GO analysis revealed that the target genes of YNJ on DCM were numerous and complex, mainly focusing on biological processes such as response to regulation to protein stability and response to oxidative stress. It was revealed that the acetone, ethyl acetate, and water extract of the rhizome of *A. asphodeloides* exhibited strong antioxidant activities (25). Moreover, the protective effect of *R. glutinosa* is associated with the increase of Mn-SOD expression and GSH level as well as the resulting scavenging effect on free radicals in cardiac muscle cells (22). *Achyranthes bidentata* polypeptides showed similar anti-oxidant and protective capacity against myocardial ischemic/reperfusion injury in rats (26). All these indicate that the cardioprotective functions of YNJ are related to alleviation of oxidative stress and the corresponding injuries induced by diabetes.

The cellular antioxidant defense system plays vital roles in the protection against oxidative damage. Nrf2, a basic leucine zipper stress-responsive transcription factor, has been recognized as a crucial mediator in ameliorating oxidative stress and enhancing cell survival by inducing the expression of multiple antioxidants and cytoprotective proteins, particularly NQO1 and HO-1. Both expression level and transcriptional activation of Nrf2, together with its target gene NQO1 level, were decreased in diabetic animals and HG-treated primary neonatal rat cardiomyocytes (27). Moreover, knocking down of Nrf2 with siRNA significantly reduced HG-injured cardiomyocyte viability (27). As a result, targeting Nrf2 signaling by pharmacological entities has been demonstrated to counteract the main pathological processes such as ventricular fibrosis and hypertrophy and then prevent the development of DCM (27, 28). Here, the KEGG pathway enrichment analysis showed that the Nrf2 signaling pathway is one of the key pathways for YNJ in the treatment of DCM. This was further validated in diabetic rats, showing that the lower expression of Nrf2 and its downstream antioxidant enzyme NQO1 was improved by YNJ administration. Moreover, several bioactive ingredients in YNJ, such as stigmasterol, diosgenin, baicalein, epiberberine, and palmatine showed high affinity with Nrf2 and/or NQO1 as indicated by molecular docking. Specifically, Timosaponin B reduced oxidative stress through the stimulation of nuclear translocation of Nrf2 and subsequent gene expression (29). In line with these previous reports, our results suggest the effects of YNJ on DCM are associated with Nrf2 signaling.

SIRT1, a NAD^+^-dependent histone deacetylase, regulates multiple biological processes, including redox hemostasis, inflammation, apoptosis, and cell metabolism. Decreased SIRT1 expression was detected in myocardial tissue of diabetic rats, along with declined antioxidant defenses and impaired cardiac morphology and function (30). Conversely, normalization of SIRT1 exhibited a protective effect against oxidative stress and hyperglycemia-induced cardiac injury by activating the Nrf2 pathway and Nrf2-dependent antioxidant genes (30, 31), or by promoting mitochondrial fusion/inhibiting mitochondrial fission and subsequent suppression of mitochondria-derived ROS production (32, 33). Moreover, SIRT1 activators may inhibit P300 and MMP-9, deacetylating histone, NF-κB, and P53 or upregulate ERK1/2, NO, and SERCA2a, resulting in increased stress resistance, thus protecting against cardiomyocyte apoptosis, fibrosis, hypertrophy, and inflammation (34). Moreover, active SIRT1 upregulated expression of NQO1, one of the most common targets of Nrf2 (35). Simultaneously, overexpression of NQO1 upregulated SIRT1 expression and activity in db/db mice by regulating the NAD^+^/NADH ratio, which was responsible for the antioxidant and antiapoptotic effects of NQO1 (36). Also, there is a direct binding between SIRT1 and NQO1 (35). Thus, the crosstalk between SIRT1 and NQO1 may be complex and they may couple in a functional module (35). In this work, we found that SIRT1 was among the key targets of YNJ to DCM. We also provide evidence for the interaction of SIRT1 and several ingredients of YNJ including stigmasterol, diosgenin, baicalein, and epiberberine. Furthermore, the decreased level of SIRT1 in diabetic myocardium was reversed by YNJ administration. These indicated that the cardioprotective effect of YNJ is related to SIRT1.

## 5 Conclusion

In summary, activation of SIRT1 and Nrf2 signaling markedly enhances expression of antioxidant enzymes, attenuates oxidative damage, and is, thus, beneficial by retarding cardiac fibrosis induced by diabetes (37). The chronic supplementation of diabetic rats with YNJ substantially prevents the development of cardiac remodeling, which is at least in part mediated by the SIRT1/Nrf2/NQO1 signaling pathway. In the future, more experiments should be performed to clarify the detailed mechanism of YNJ. Despite this, our findings identify YNJ as a potential strategy for the prevention and treatment of DCM that deserves further investigation.

## Data availability statement

Publicly available datasets were analyzed in this study. This data can be found here: NCBI: GSE4745.

## Author contributions

FW conceived and designed the study and provided funding support. WW performed network pharmacology research, molecular docking, and animal experiments and wrote the first draft of manuscript. RL helped analyze the data and prepare the manuscript. YZ revised the manuscript and provided funding support. LW, YT, BD, and ST helped conduct the experiment. All authors contributed to the article and approved the submitted version.
